# Effect of neuroinflammation on the progression of Alzheimer’s disease and its significant ramifications for novel anti-inflammatory treatments

**DOI:** 10.1016/j.ibneur.2025.05.005

**Published:** 2025-05-22

**Authors:** Pritam Kamila, Koyel Kar, Sailee Chowdhury, Priyanka Chakraborty, Ria Dutta, Sowmiya S, Ankul Singh S, Bhupendra Gopalbhai Prajapati

**Affiliations:** a BCDA College of Pharmacy & Technology, Department of Pharmaceutical Chemistry, 78/1- Jessore Road (South), Hridaypur, Kolkata, West Bengal 700127, India; b Department of Pharmacology, Faculty of Pharmacy, Dr. M.G.R Educational and Research Institute, Velappanchavadi, Chennai, Tamil Nadu 600077, India; c Shree S.K. Patel College of Pharmaceutical Education & Research, Ganpat University, Kherva, Mehsana, Gujarat 384012, India; d Centre for Research Impact & Outcome, Chitkara College of Pharmacy, Chitkara University, Rajpura, Punjab 140401, India; e Department of Industrial Pharmacy, Faculty of Pharmacy, Silpakorn University, Nakhon Pathom 73000, Thailand

**Keywords:** Alzheimer’s disease, Neuroinflammation, Microglia, Cytokines, Anti-inflammatory therapy, Glial cells

## Abstract

Alzheimer’s disease (AD) is increasingly recognized as a disorder not solely of amyloid and tau accumulation but also of chronic immune dysregulation. Emerging evidence highlights the critical role of neuroinflammation, characterized by sustained activation of microglia and astrocytes, cytokine release, and inflammasome activation in accelerating AD progression. Genome-wide studies have further identified key inflammatory genes and immune pathways associated with increased disease risk. This review critically evaluates the mechanistic underpinnings of neuroinflammation in AD, focusing on glial cell behavior, immune signaling, and their contribution to neuronal dysfunction. Importantly, the review highlights recent advances in anti-inflammatory therapeutic approaches, including modulators of IL-1β, TNF-α, TREM2, and CB2 pathways. By integrating mechanistic and therapeutic insights, this work underscores the potential of immunomodulatory strategies as viable interventions in AD and provides a novel framework for future research in targeted anti-neuroinflammatory treatments.

## Graphical Abstract

**Figure:**
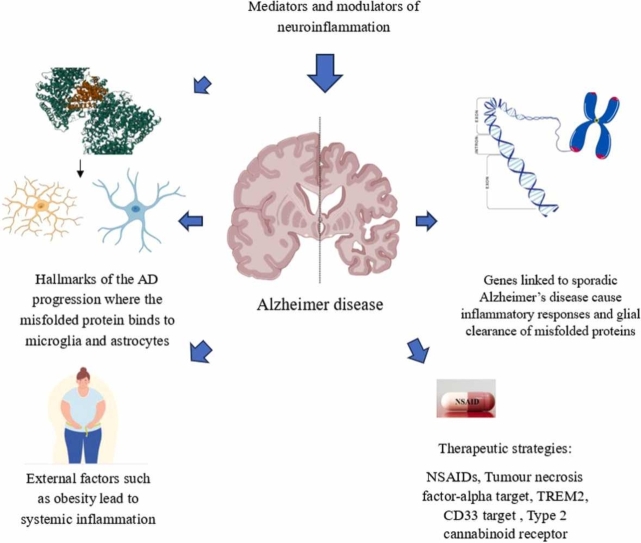

## 1 Introduction

Traditionally viewed as separate disciplines, immunology and neurobiology are increasingly intersecting, particularly in the context of neurodegenerative diseases. Alzheimer’s disease (AD), a progressive neurological disorder and the leading cause of dementia among the elderly, exemplifies this convergence. With the global dementia burden projected to reach 131.5 million by 2050 (Prince et al., 2016). Currently, no therapy can completely halt the progression of AD, and only a few available medications demonstrate modest potential in slowing disease progression. Although amyloid-β (Aβ) peptide accumulation is considered a critical early event in AD pathogenesis, the underlying mechanisms remain unclear. Despite numerous ongoing clinical trials, most Alzheimer's treatments have failed to achieve significant efficacy. Given the emergence of anti-inflammatory agents as promising therapeutic candidates, this review provides an updated perspective on the role of neuroinflammation in AD progression (Cummings et al., 2021).

Recent advances point to chronic neuroinflammation as a key driver in AD pathogenesis rather than a mere byproduct (Toader et al., 2024). Microglia and astrocytes, the primary immune cells of the central nervous system, play dual roles in neuroprotection and neurodegeneration (Colonna and Butovsky, 2017). Their sustained activation leads to the release of pro-inflammatory mediators, oxidative stress, and synaptic dysfunction (Li et al., 2025, Yang et al., 2025) Furthermore, genetic studies implicate variants in immune-related genes such as TREM2 and APOE4 in modulating the inflammatory response and influencing disease susceptibility (Zhang et al., 2025).

This review explores the central role of neuroinflammation in AD progression and its therapeutic implications. Particular emphasis is placed on cellular and molecular mediators of inflammation and their potential as novel drug targets. By critically examining the inflammatory mechanisms underpinning AD, we aim to inform the development of targeted anti-inflammatory therapies and shift the therapeutic paradigm beyond conventional amyloid-centric approaches.

## 2 Pathogenesis and hypotheses of Alzheimer’s disease

**Fig. 1:**
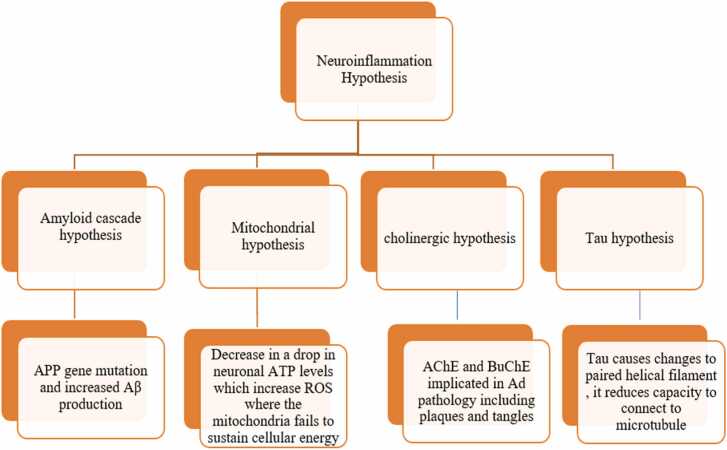
Illustrates how amyloid, tau, cholinergic, and mitochondrial dysfunctions converge on neuroinflammation as a central pathological mechanism in Alzheimer’s disease.

AD is a neurological ailment characterized by a gradual loss of memory, cognitive function, and behavioral abilities. The etiology of AD is complex, involving a combination of environmental, genetic, and behavioral factors that play various roles in the progression of the disease (Monteiro et al., 2023). Several hypotheses have been proposed to explain the pathological mechanisms of AD. These hypotheses emphasize the disease's complex and multifaceted nature and help define a roadmap for exploring potential therapeutic options. While the Aβ cascade hypothesis has received the greatest attention (Kepp et al., 2023) Emerging theories such as tau pathology, neuroinflammation, and mitochondrial dysfunction further highlight the multifactorial nature of AD (Fig. 1) (Pérez et al., 2018, Tracy et al., 2022, Chen and Yu, 2023). Having outlined these key hypotheses, we now focus on the neuroinflammatory mechanisms and how they integrate with other pathogenic processes.

### 2.1 Neuroinflammation hypothesis

The term "neuroinflammation" describes the central nervous system’s highly complex response to stimuli such as infections, trauma, and neurodegenerative diseases. This cellular immune response involves the production of reactive oxygen and nitrogen species, stimulation of glial cells, and release of inflammatory mediators. Chronic or dysregulated inflammation can lead to pathological states like Parkinson’s disease, Alzheimer’s, Multiple Sclerosis, and other neurodegenerative disorders involving widespread neuronal damage. Neuroinflammation is crucial in protecting the brain from pathogens (Adamu et al., 2024); however, the outcome is heavily influenced by the response of key cellular participants, including microglia and astrocytes. These cells release pro- and anti-inflammatory cytokines, growth factors, and bioenergetic mediators that contribute to brain homeostasis, cognitive function, and neuronal survival (Dubois et al., 2016). Although the blood-brain barrier (BBB) normally maintains selectivity, this can be disrupted by aging or disease. Senescent microglia and astrocytes exhibit a persistently hyperactivated state, remaining unresponsive to typical resolution signals. These cells adopt a senescence-associated secretory phenotype (SASP), continuously releasing pro-inflammatory proteins and biochemical mediators. This chronic activation contributes to neurodegeneration, cognitive decline, and increased mortality through progressive neuronal damage and impaired neuroprotection (Onyango et al., 2016). Fig. 1 illustrates how amyloid, tau, cholinergic, and mitochondrial dysfunctions converge on neuroinflammation as a central pathological mechanism in Alzheimer’s disease.

#### 2.1.1 Amyloid cascade hypothesis

**Fig. 2:**
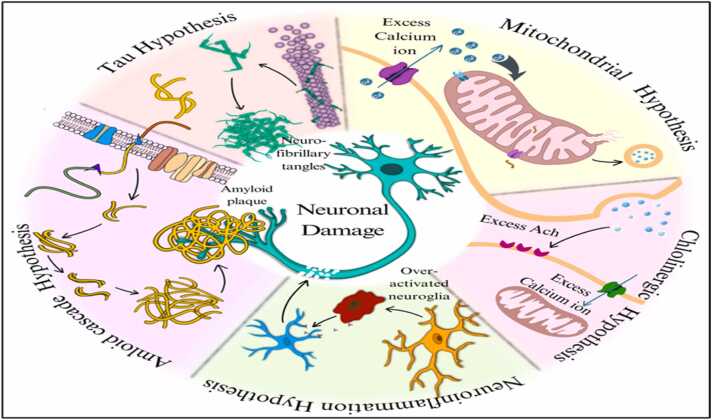
Different Hypotheses of AD.

This widely accepted hypothesis explains the formation of senile plaques and the accumulation of Aβ oligomers, key pathological features of AD. The Aβ hypothesis, also called the amyloid cascade hypothesis, proposes that Aβ is cleaved from amyloid precursor protein (APP) by β- and γ-secretases and released extracellularly (Fig. 2). In healthy individuals, Aβ is rapidly degraded or cleared. However, in aging or disease, the capacity to degrade Aβ diminishes, leading to its accumulation. Among its isoforms, Aβ42 (more hydrophobic than Aβ40) is more prone to aggregation, resulting in the formation of insoluble fibrils and senile plaques (Karlawish et al., 2017). These plaques are associated with neurotoxicity, tau pathology, and neuronal degeneration. The hypothesis is supported by the association between APP gene mutations and increased Aβ production (Hardy and Selkoe, 2002).

#### 2.1.2 Mitochondrial hypothesis

In AD, mitochondrial deficient functionality is evident in a drop in neuronal ATP levels, which is coupled with ROS overproduction and suggests that mitochondria may fail to sustain cellular energy. ROS are defined as chemically reactive oxygen-free radicals with no radical derivatives of oxygen. Either increased ROS engendering or a defective antioxidant system can shift the cell's redox balance to an oxidative state. Because of the higher energy requirements of neuroglia and neurons, the brain consumes a significant quantity of ATP. Because the central nervous system (CNS) lacks an energy store (such as fat or glucose), brain cells must constantly create ATP to manage neuronal activity (Khatri and Man, 2013). Importantly, oxidative damage to the promoter of the gene that encodes the mitochondrial ATP synthase component results in lower amounts of the related protein, which leads to decreased ATP generation, nuclear DNA damage to vulnerable genes, and function loss (Reed et al., 2008). The dwindle in AD brain bioenergetics caused by deficient functionality of mitochondria is a clear biomarker that reveals the endurance of the disease before symptoms manifest. Reduced mitochondrial bioenergetics, metabolic enzyme expression, and activity, and also brain glucose metabolism, together with elevated oxidative stress with Aβ deposits, are the typical characteristics of mitochondrial failure. These mitochondrial deficient functionalities are connected with AD's prodromal stage (Belenguer et al., 2019).

#### 2.1.3 Cholinergic hypothesis

The cholinergic hypothesis proposes that raising the synaptic level of ACh or allosteric synchronization of postsynaptic cholinergic receptors with selective agonists may boost the reliability of cholinergic synaptic activation or number downregulation. The second option is best met by activating or modifying either the M1 type receptor or an important nicotinic receptor subtype. Both approaches have been clinically utilized (Fisher, 2012). M1 receptors play a contribution to learning and building memory processes, as evidenced by effects in various animals as models, and are compromised at various junctures of AD. Acetylcholinesterase (AChE) and butyrylcholinesterase (BuChE) are the two utmost significant cholinesterases in mammalian brains, with genetics, structure, and kinetics that differ. AChE is mostly found in neuronal synapses and blood, whereas BuChE is found around neuroglia and neurons withheld in the human brain, likewise tangles and neuritic plaques in Alzheimer's patients (DeTure and Dickson, 2019).

#### 2.1.4 Tau hypothesis

Tau is an important protein tied to microtubules that helps to maintain tubulin assembly stability. The human tau gene is present on the chromosome. Tau's biological activity is governed by its phosphorylation level in the cerebral region-stem. Tau typically includes a 2–4:1 ratio of phosphate to protein moles, while the AD brain contains more phosphates (Kopke et al., 1993). In AD, excessive or hyperphosphorylation of the Tau causes it to change from normal adult Tau to a paired helical filament (PHF-tau), reducing its capacity to connect to microtubules (Bejanin et al., 2017). chromosomal alterations cause tau to clump and form an insoluble structure, as antagonistic to their typical soluble structure (Mohandas et al., 2009).

## 3 Mechanisms and mediators of neuroinflammation in AD

Various peripheral white blood cells (WBCs), including T cells, NK cells, B cells, and neutrophils, significantly influence the progression of AD. Neutrophils, when activated, can adhere to the vascular endothelium, leading to aggregation and potentially disrupting cerebral blood flow. By releasing pro-inflammatory cytokines and neutrophil extracellular traps (NETs), they exacerbate neuroinflammation in brain regions compromised by a weakened BBB (Zenaro et al., 2015). T cells indirectly facilitate disease advancement by affecting the functional dynamics of neuroglia (Ito et al., 2019). In contrast, B cells produce cytokines and antibodies that impact disease continuation and influence behavioral responses (Xiong et al., 2021). NK cells, on the other hand, interact with neuroglia to produce immune-regulatory molecules, thereby undergoing neurodegeneration (Zhang et al., 2020).

### 3.1 Mediators and modulators of neuroinflammation

Numerous mediators and modulators of neuroinflammation, including chemokines, cytokines, etc, are dysregulated in the inflammatory sequence mechanism of AD (Table 1).

**Table 1:** Different modulators and mediators of AD.

Name of modulators	Role of the modulators	References
Cytokines	Astrocytes along with microglia, release cytokines. They are interrelated with the escalation of IL−1b and IL−12. Patients with AD have elevated levels of IL−1b and IL−12.	(Ogunmokun et al., 2021b)
Chemokines	Chemokines are integrated into the reactivation of neuroglia cells. Neurons express CX3CL1while microglia mostly express CX3CR1. CX3CL1/CX3CR1 gets complicated in the AD patients.	(Kang et al., 2021)
Other mediators	Three distinct nitric oxide synthases (NOS) are either neuroinhibitory or neurodefensive. NO can cause protein nitrosylation, long-term potentiation impairment, or mitochondrial respiration suppression.	(Picón-Pagès et al., 2019)

#### 3.1.1 Cytokines

**Fig. 3:**
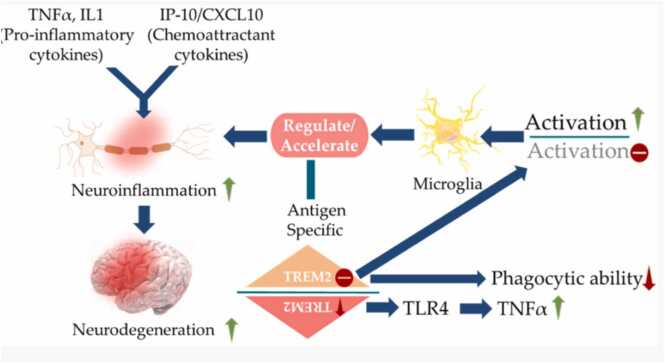
Role of cytokines in Alzheimer's disease (Ogunmokun et al., 2021a).

Neuroinflammation, defined as an acute or chronic inflammatory response within the central nervous system (CNS), is frequently associated with increased levels of proinflammatory cytokines such as IL-1β, IL-6, IL-18, and TNF-α. These cytokines disrupt synaptic function, contribute to neuronal damage, and reduce neurogenesis. Neuroglia, a diverse group of non-neuronal cells essential for CNS homeostasis, was originally described as "connective tissue that binds nervous elements together (Luca et al., 2018). Cytokines like IL-1β and IL-12 play critical roles in AD pathogenesis. Elevated serum IL-1β is often observed in patients with mild cognitive impairment considered a prodromal stage of AD (Forlenza et al., 2009). IL-1β polymorphisms, particularly when coexisting with the APOE-ε4 allele, are associated with increased IL-1β levels and sleep disturbances in AD patients (Graybeal et al., 2014). IL-12 is also implicated in the disease's immune axis. In a Han Chinese population, a polymorphism in IL-12 was linked to increased AD risk (Zhu et al., 2014). Vom Berg *et al*. hypothesized that targeting the IL-12/IL-23 signaling axis by blocking the IL-12p40 subunit could reduce AD pathology (vom Berg et al., 2012a). Correspondingly, higher IL-12p40 concentrations have been reported in the CSF of AD patients. Nonetheless, IL-10 deletion in APP/PS1 mice improved behavior and synaptic integrity, suggesting an unexpected neuroprotective role of proinflammatory balance **(**vom Berg et al., 2012b**).** Overexpression of IL-10 using adeno-associated viruses (AAVs) in transgenic mice led to increased Aβ accumulation, behavioral abnormalities, and impaired microglial phagocytosis of Aβ. Similarly, TGF-β, another important cytokine, is elevated in the CSF of AD patients along with Aβ plaques (Chakrabarty et al., 2015). Fig. 3 illustrates the role of cytokines in Alzheimer's disease.

#### 3.1.2 Chemokines

Chemokines, a subclass of cytokines, play a key role in leukocyte recruitment, chemotaxis, and immune cell regulation during inflammation. These small (8–14 kDa) proteins are grouped based on conserved cysteine residues into four main subfamilies: CXC (α-chemokines), CC (β-chemokines), CX3C (δ-chemokine, e.g., CX3CL1/fractalkine), and XC (γ-chemokines) (Domingues et al., 2017, Wojcieszak et al., 2022). According to Martin and Delarasse, chemokines are crucial for Aβ plaque formation and neurofibrillary tangle development in AD. For instance, chemokine receptors such as CXCR2 and CCR3 promote Aβ synthesis, while CX3CR1 and CCR2 may reduce its deposition. Additionally, chemokines like MCP-1 and receptors including CX3CR1 and CCR3 are implicated in tau phosphorylation, further advancing AD pathology (Wang et al., 2023, Kim et al., 2008). Elevated CX3CL1 levels in tau-damaged neurons and reduced expression in APP-transgenic mice imply that the same chemokine may exert different effects depending on the disease stage. Moreover, higher CCL2 levels in early-stage AD correlate with faster cognitive decline, suggesting a pathogenic role in initial disease progression (Westin et al., 2012).

#### 3.1.3 Other inflammatory mediators

Nitric oxide (NO), a gaseous free radical belonging to the reactive nitrogen species (RNS) family, is produced by nitric oxide synthase (NOS) enzymes from L-arginine and oxygen (Spiers et al., 2019). In the CNS, NO plays both neuroprotective and neurotoxic roles, depending on its concentration, the NOS isoform involved, and the disease stage. Physiologically, NO supports synaptic plasticity modulates neuronal excitability, and enhances cognitive functions through the regulation of potassium channels like Kv7 and Kv2 (Balez and Ooi, 2016). However, excessive NO especially from inducible NOS (iNOS) can result in nitrosative stress, leading to mitochondrial dysfunction, DNA damage, and the generation of peroxynitrite, a potent oxidant implicated in neuronal injury in AD (Azargoonjahromi, 2023). Vascular dysfunction is another contributor to AD, as decreased perfusion in regions such as the frontal cortex and mesial temporal lobes has been observed. Reduced NO bioavailability in cerebral vessels may impair vasomotor tone, promoting neurovascular decline and cognitive impairment (Venturelli et al., 2018).

### 3.2 Factors facilitating neuroinflammation

**Fig. 4:**
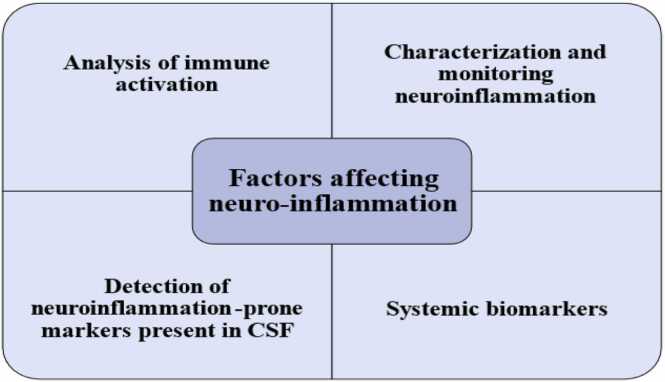
Factors affecting neuroinflammation.

The accumulation of Aβ alone can trigger an inflammatory immune response, even a decade before clinical symptoms manifest. Thus, modifiable risk factors, such as systemic inflammation, obesity, and cerebral trauma can exacerbate neuroinflammation (Fig. 4). Neurodegenerative disorders share common hallmarks, including regional brain vulnerability, chronic inflammation, and the accumulation of misfolded proteins (Shi et al., 2017). While the innate immune response helps eliminate pathogens and restore homeostasis, continuous activation due to endogenous factors like protein aggregates, environmental insults, or genetic predispositions (APOE4 and PGRN mutations) may lead to sustained inflammation. In this context, a lack of specialized pro-resolving mediators (SPMs) that facilitate inflammation resolution is considered a key factor in persistent neuroinflammation (Hanisch and Kettenmann, 2007)**.**

#### 3.2.1 Analysis of immune activation

Selective vulnerability of specific brain regions in AD is linked to their neuronal response to toxic insults, such as misfolded proteins. Chronic microglial activation and increased leukocyte infiltration across the BBB contribute to progressive neuronal damage. Furthermore, the innate immune network interacts with adaptive immunity via antigen presentation and signaling. Astrocytes and microglia, the primary immune cells in the CNS are tightly involved in the pathological processes of AD (Szmydynger-Chodobska et al., 2009). Microglia, in particular, mediate surveillance, debris clearance, and synaptic maintenance. In their resting state, they show low MHC expression, but when activated, assume an amoeboid shape with increased MHC and co-stimulatory molecule expression, enabling interaction with T cells and propagation of inflammation (Lécuyer et al., 2016). Pro-inflammatory molecules like tumor necrosis factor-alpha (TNFα), IL-1β, interferon-gamma (IFN-γ), chemokines, and reactive nitrogen and oxygen species (RNS/ROS) are released in the latter state of microglial in conjunction with morphological changes. These molecules encourage peripheral leukocyte diapedesis through the BBB, which further fuels the local harmful inflammatory response. Microglia can adopt an alternate activation pathway with their pro-inflammatory pattern (Novoa et al., 2022)**.**

#### 3.2.2 Characterization and monitoring of neuroinflammation

The distinguishing markers of immune-response cells still need to be proven as a useful diagnostic or surveillance tool for AD, despite mounting evidence that inflammation influences the cytology of the disease. However, recent research of postmortem acute AD brains using gene-expression analysis revealed new information on an immunological and microglial cell network that is dominated by genes tied to phagocytosis. These findings will be the subject of future research, coupled with novel studies of inflammation-related biomarkers in external blood, CSF, or distinctly in the cerebral region via imaging. The pinpointing of prodromal AD phases by inflammatory biomarkers will be a main component of this investigation (Kreisl et al., 2013).

#### 3.2.3 Detection of neuroinflammation-prone markers present in CSF

Numerous research studies have examined the cytokine levels of pro-inflammasome proteins and anti-inflammatory types in CSF throughout the previous 25 years. These studies' findings are frequently debatable. However, different signs that the sampling time point, the disease period, is a pivotal element of the investigation. Elevated CSF cytokines have been recognized in certain studies as indicators of the pace of cognitive loss and disease development or as elements that alter the escalation of MCI to AD. Achieving a comprehensive assessment of cytokine management for AD requires harmonized approaches, detailed patient characterization, and long-term sampling, akin to imaging consortia (Galimberti et al., 2008).

#### 3.2.4 Systemic biomarkers

Numerous studies revealed that there is a complex interaction between the cerebral cortex and the systemic surroundings. Peripheral immune-response cells and secreted signaling proteins that interact with the brain have been connected to neuroinflammation processes in general. Although cells may penetrate cerebral tissue in these interactions, soluble signaling molecules found in the systemic environment, such as blood-borne factors, probably mediate many more. These elements can be adjuvant in reviving neuronal regrowth in mice's aged brains or age-dependently inhibit or stimulate adult neurogenesis (Czirr and Wyss-Coray, 2012). Scientists have looked for cellular alterations in blood tied to neurodegenerative diseases to pinpoint these causes. Numerous proteomic methods are possibly used for identifying blood-based biomarkers. Trophic components, like cytokines and brain-derived neurotrophic factor (BDNF), have been the most often used biomarkers (Chowdhury et al., 2024). Protein assessments in samples of plasma from participants of the AD neurological examination experiment were closely related to "communicate," resulting in protein profiles tied to patients who advanced from moderate mental retardation to AD.

## 4 Neuroimmune dynamics in AD progression

Nearly 15 % of those 65 and older and over 25 % of people 85 and older globally are plagued by AD, having dementia (about 75 % of cases) (Kiraly et al., 2023a). The buildup of Aβ along with tau deposits and fibrils is interrelated with the steady deterioration of memory and, likewise cognitive capacity. In brief, units of peptides of Aβ of different sizes are produced by the fragmentation of amyloid precursor proteins (APP). Large plaques and insoluble polymers can be created when Aβ oligomers combine with single-peptide monomers. Now widely admired is that Aβ oligomers cause the utmost serious pathogenic harm to neurons (Selkoe, 2008; Wang and Mandelkow, 2016). Positron emission tomography (PET) imaging and cerebrospinal fluid (CSF) measures of Aβ, occasionally in conjunction with studies of CSF tau forms, are currently the gold standards for diagnosing AD (Brand et al., 2022). However, clinical trials attempting to remove senile plaques from AD patients' brains revealed conflicting outcomes: in specific instances, the volume of protein accretions dropped, but the removal did not enhance cognitive function (Lannfelt et al., 2014). Nonetheless, current research indicates that monoclonal antibody therapies, using drugs such as lecanemab, aducanumab, and, most recently, donanemab, can eliminate Aβ buildup to 90 % and considerably decrease the disease's continuation in the beginning of AD. Compared to 29 % of patients who took a placebo, 47 % of patients did not experience any worsening of mental consciousness decline. However, amyloid-related imaging abnormalities (ARIA) continue to be fatal for the significant adverse impact of antibody therapy treatment. Nearly 30 % of individuals in the Phase III trial got ARIA, and four of them passed away from the disease (Reardon, 2023).

Given the pivotal role that neuroinflammatory processes play in exacerbating AD pathology including glial activation, cytokine dysregulation, and impaired phagocytic clearance there is a pressing need to explore interventions that directly modulate these immune responses. Recent advances in immunoneurology have therefore led to the identification of several promising therapeutic targets within the neuroinflammatory cascade. The following section outlines these emerging strategies and their potential to reshape AD treatment paradigms.

### 4.1 Impact of astrocytes, neuroglia, and cell mediators in AD neuroinflammation

All primary mechanisms that protect brain homeostasis have evolved to counteract neuroinflammation. It serves a highly conserved role in defending and restoring synaptic function following viral injury. However, the diverse cellular responses from various system participants—including neurons, astrocytes, and neuroglia—significantly influence physiological outcomes. Neuroinflammatory feedback and the leakage of blood cells through a compromised BBB can exacerbate the damage. When this persists, the condition is regarded as chronic and is likely uncontrollable without therapeutic intervention (Onyango et al., 2021). Proinflammatory mediators secreted in the impaired CNS cause neurotoxicity and reduction of neurogenesis (Lyman et al., 2014). The overexpression of IL-1β and TNF-α, mediated through prostaglandin E2, results in synaptic loss (Mishra et al., 2012). Moreover, coagulation factors, proteases, pentraxins, and other molecular agents are significant contributors to neural degeneration and functional impairment (Yang, 2019). As a major support system within the CNS, astrocytes play a vital role in maintaining BBB integrity, regulating neurotransmitter balance, and supporting both newly formed and mature synapses (Leng and Edison, 2021a; Liddelow et al., 2017a). The glial fibrillary acidic protein (GFAP), their principal structural protein, has become a biomarker for identifying reactive astrocytes. It remains unclear whether astrocytes can be divided into distinct subtypes or if they exist along a dynamic spectrum representing dual phenotypic traits (Leng and Edison, 2021b). However, the functional impact of reactive astrocytes remains under debate. Notably, high levels of pro-inflammasome proteins have been detected in astrocytes within postmortem brain tissue from AD patients, suggesting pathological activation of A1 astrocytes (Liddelow et al., 2017b). Furthermore, evidence indicates that these reactive A1 astrocytes disrupt cerebral blood flow and compromise BBB function, contributing to both the onset and progression of neurodegenerative diseases (Leng and Edison, 2021a).

Microglia, the brain's primary innate immune cells, are essential for phagocytosing pathogens and cellular debris, and for secreting factors that maintain the homeostasis of the neural environment. They also play a critical role in modulating synaptic plasticity (Ji et al., 2013). Contemporary research suggests that elevated cytokine levels in cerebrospinal fluid (CSF) may impair microglial clearance of Aβ oligomers (Hickman et al., 2008, Guerreiro et al., 2013). Evidence implies that TREM2 encourages phagocytosis expressed in neuroglia (Hickman et al., 2013). When pathologically overstimulated, microglia may undergo morphological changes, adopting amoeboid forms with reduced processes and a narrowed surveillance field. The extent and persistence of environmental insults influence the morphological and functional shifts in microglia, correlating with the degree of neural damage (Leng and Edison, 2021b, Sanchez-Mejias et al., 2016). Microglia are traditionally classified using the nomenclature M1 for the inflammation-causing characteristic and M2 for the anti-inflammatory component (Leng and Edison, 2021c). However, like astrocytes, microglial activation may be better represented as a continuum of phenotypic states rather than discrete categories. Upregulation of proteins such as apolipoprotein E (APOE), TREM2, and TYROBP is typically associated with the activation of microglial states. During the early stages of neuroinflammation, genes involved in cell proliferation become increasingly expressed (Keren-Shaul et al., 2017; Mathys et al., 2017). Microglial cells, which are the central nervous system's intrinsic macrophages, are in charge of keeping an eye on and reacting to insults and injuries in the surrounding brain. Upon activation, they play a key role in the brain’s natural defense system by attempting to clear misfolded protein fibrils, including Aβ, through phagocytosis. They surround and attempt to degrade abnormally accumulated proteins. A chronic and ultimately inefficient microglial activation is seen in Alzheimer's disease and other neurodegenerative diseases (Dani et al., 2018). According to recent research, the cerebrospinal fluid's (CSF) elevated cytokine levels may impair microglia's ability to absorb Aβ. Additionally, according to recent data, a rare mutation of the TREM2 extracellular unit raises the risk of developing AD to a degree comparable to that of the apolipoprotein unit apoEα4 (Li et al., 2021).

Microglial cells have high levels of TREM2, which has been demonstrated to promote phagocytosis (Kiraly et al., 2023a). TREM2 and transient receptor potential melastatin-related 2 (TRPM2) channels have emerged as promising molecular targets for microglia-based pharmaceutical therapies (Malko et al., 2019)**.** TRPM2 mediates calcium influx, which in turn activates NF-κB and facilitates the assembly of the NLRP3 (NOD-, LRR-, and pyrin domain-containing protein 3) inflammasome. Apoptosis-associated speck-like proteins serve as a scaffold for Aβ peptide aggregation, thereby triggering NLRP3 activation. These aggregates can then spread to adjacent cells, promoting Aβ formation. Pharmacological blockade of this pathway could potentially slow AD progression. TREM2 is activated via the immunoreceptor tyrosine-based activation motif (ITAM) pathway, rendering microglia reactive (Venegas et al., 2017)**.** One downstream signaling route involves spleen tyrosine kinase (SYK) and the transmembrane adaptor DAP12. SYK phosphorylates various downstream effectors—including the PI3K-AKT-mTOR and PLCγ2-Ca²⁺ cascades, leading to GSK3β inhibition and promotion of microglial survival and proliferation. Recent studies reveal that SYK deficiency impairs microglial phagocytosis of Aβ, resulting in increased neurotoxicity. Notably, Aβ accumulation was equally severe in SYK-deficient mice and TREM2 knockout models (Wang and Colonna, 2023), suggesting that TREM2 and SYK have parallel roles in AD pathology. Therefore, targeting the TREM2-YINM/ITAM-SYK signaling axis with small molecules or immune-based therapies represents a compelling avenue for AD treatment. Through adaptors DAP10 (YINM) or DAP12 (ITAM), TREM2 initiates intracellular signaling; while YINM directly recruits PI3K, ITAM does so indirectly via SYK. Both pathways modulate essential cellular functions by activating mTOR and inhibiting GSK3β through AKT signaling (Li et al., 2023). Currently, TREM2 is evaluated for antibody-mediated treatment (Wang et al., 2020a).

However, optimizing pharmacological strategies to regulate TREM2 signaling in a disease-state-specific manner remains crucial. One study using an AD animal model showed that increased TREM2 expression correlated positively with genes linked to phagocytosis and negatively with immune-related genes, resulting in a net neuroprotective effect (Lee et al., 2018). Conversely, other studies found that TREM2 activation elevated neuroinflammation and APOE-dependent signaling, disrupting microglial homeostasis (Krasemann et al., 2017)**.** TREM2 thus acts as a central regulator of downstream mechanisms governing microglial phenotypic shifts and the neuroglial response cycle. These findings underscore that experimental design, disease model, and pathological context can significantly affect the interpretation of outcomes. Careful consideration and a nuanced understanding of these variables are essential for translating preclinical results into effective clinical therapies.

### 4.2 Type 2 cannabinoid receptor as a target for neuroprotection

The cannabinoid receptor (CB) family includes a pair of duplicated metabotropic receptors: CB1 and CB2. While CB2 is predominantly expressed in the peripheral nervous system (PNS), CB1 is primarily concentrated in the brain (Núñez et al., 2004). However, reactive neuroglia associated with multiple sclerosis, HIV encephalitis, Huntington's disease, and Alzheimer's disease (AD) express elevated levels of CB2 receptors (Benito et al., 2008). Experimental studies demonstrate that neuroglial activation occurs in mouse models of AD, accompanied by increased CB2 expression and upregulation of pro-inflammasome proteins, which contribute to neuronal damage (Bie et al., 2018). Triggering the CB2 receptor might reduce neuroinflammation-prone responses in numerous CNS disorders, including AD (Magham et al., 2021). In C6 rat astroglioma cells treated with Aβ fibrils, CB2 expression is significantly upregulated, indicating a role in Aβ-associated diseases (Esposito et al., 2007).

Protein kinases may also regulate microglial phenotypes, potentially preventing neuronal destruction and limiting synaptic loss via the release of proinflammatory cytokines (Ghosh et al., 2016)**.** Furthermore, the production of nitric oxide (NO) and chemokines by lipopolysaccharide (LPS)--activated microglia is suppressed through β-arrestin recruitment and the use of intracellular Ca²⁺ chelators (Stebbing et al., 2015). Reducing ceramide accumulation enhances cellular viability and downregulates the extracellular signal-regulated kinase (ERK) cascade pathway (Zou and Kumar, 2018). Toll-like receptors (TLRs) play crucial roles in phagocytosis and absorption (Ruiz de Martín Esteban et al., 2022a). Importantly, promoting a neuroprotective glial phenotype appears to mitigate brain atrophy—a hallmark of neurodegenerative disorders such as AD—by protecting neurons from degeneration (Guo et al., 2022)**.** Data from β-arrestin-evaluated studies indicate that the lead compound NTRX-07 [1-((3-benzyl-3-methyl-2,3-dihydro-1-benzofuran-6-yl) carbonyl) piperidine] acts through both canonical (adenylate cyclase inhibition) and noncanonical (CB2-mediated) pathways to exert its therapeutic effects. In animal models, elevated levels of hyperphosphorylated tau compromise hippocampal function and are associated with increased neuroinflammation, driven by excessive cytokine production (Dionisio-Santos et al., 2019)**.** CB2 receptor activation has been shown to reduce neuroinflammation and glial activation, leading to enhanced glutamatergic signaling, improved hippocampal plasticity, reduced Aβ plaque burden, and better memory performance in Morris water maze tests (Wu et al., 2017a)**.** Administration of NTRX-07 also reduced IBA1 (ionized calcium-binding adaptor molecule 1) immunoreactivity and enhanced CB2 receptor expression in the entorhinal cortex (Bie et al., 2018). Statistical significance was determined using the Student–Newman–Keuls test and various ANOVA models. This suggests that NTRX-07 restores functional memory in AD mouse models through anti-inflammatory and neuroprotective mechanisms (Wu et al., 2013, Wu et al., 2017b).

## 5 Therapeutic strategies targeting neuroinflammation in AD

### 5.1 Therapeutic targets of neuroinflammation and treatment strategies in AD

Innovative therapeutic approaches have gained attention due to the persistent failure of numerous anti-Aβ or anti-tau strategies, despite substantial investments by multinational pharmaceutical companies and research institutes. This challenge persists even after the recent US FDA approval of aducanumab for AD treatment (Lista et al., 2024). The urgent need for novel and effective interventions is underscored by the growing prevalence of AD and its complex pathophysiology. Although the exact cause of AD remains unclear, it is believed to arise from an interplay of genetic and lifestyle factors (Shue et al., 2024, Chen et al., 2024).

#### 5.1.1 Tumor necrosis factor-alpha target and as a therapeutic agent

Building on the growing understanding of neuroinflammation’s role in AD, researchers have shifted focus toward immunomodulatory therapies. These therapeutic strategies aim to address the failure of traditional Aβ and tau-targeting agents by intervening earlier in the inflammatory cascade. One important pro-inflammatory cytokine implicated in AD pathology is tumor necrosis factor-alpha (TNF-α), primarily secreted by activated microglia (Decourt et al., 2017, MacEwan, 2002). TNF-α exerts its effects via two receptors: TNFR1, which promotes pro-inflammatory responses, and TNFR2, which has neuroprotective and immunomodulatory roles. The transmembrane form of TNF-α (tmTNF) can activate both receptors, whereas soluble TNF (sTNF) primarily signals through TNFR1. Modulating TNF/TNFR signaling—either at the ligand or receptor level—has emerged as a potential strategy (Detrait et al., 2014). Recent studies have focused on selective activation of TNFR2 while inhibiting sTNF/TNFR1 signaling. A study investigated the therapeutic potential of a TNFR2 agonist (EHD2-scTNFR2) and a TNFR1 antagonist (ATROSAB) in mice with NMDA-induced neurotoxicity. Both agents were found to restore cholinergic function, reduce microglial activation at lesion sites, and protect cholinergic neurons from excitotoxicity (Dong et al., 2016; Sun et al., 2024).

#### 5.1.2 Targeting TREM2 (triggering receptor expressed on myeloid cells 2) as a treatment option

Soluble TREM2 (sTREM2) has emerged as a neuroinflammation-associated biomarker relevant to AD pathology (Bekris et al., 2018). In 5 ×FAD transgenic mice expressing the human R47H TREM2 variant, microglial recruitment and activation near Aβ plaques were impaired (Song et al., 2018)**.** By contrast, in mice with the common variant of TREM2, sTREM2 was detectable in plaques and neurons. Gene transfer of recombinant sTREM2 via adeno-associated virus was shown to reduce plaque accumulation and improve spatial memory and long-term potentiation. In these models, sTREM2 promoted Aβ clearance, reduced Aβ deposition, and enhanced microglial activity (Zhong et al., 2019; Price et al., 2020). Treatment with AL002c, a TREM2 agonistic antibody, led to increased microglial proliferation and activity in R47H mice compared to common variant mice. AL002c was found to modulate behavior and reduce neuroinflammatory responses, particularly in areas with branched Aβ fibrils and Aβ aggregates, which contribute to axonal damage and neurite dystrophy (Wang et al., 2020b).

#### 5.1.3 CD33 target and as a therapeutic agent

*In vitro* studies have demonstrated that wild-type CD33 (WT-CD33) expression in BV2 microglia reduces the uptake and degradation of Aβ42. Moreover, in APPSwe/PS1ΔE9/CD33 knockout mice, brain Aβ plaque burden and soluble Aβ42 levels were significantly lower compared to mice expressing CD33, highlighting its potential as a therapeutic target (Lim et al., 2000).

#### 5.1.4 Non-steroidal anti-inflammatory drugs

NSAIDs have been evaluated in multiple AD mouse models with variable outcomes. For instance, long-term oral administration of ibuprofen reduced cortical Aβ levels, plaque burden, and neuroinflammation in Tg2576 mice. However, Jantzen et al. found no significant reduction in Aβ load in the same model using similar treatment conditions (Jantzen et al., 2002). Likewise, selective COX-2 inhibitors such as celecoxib and mesulide did not show appreciable effects on Aβ levels in Tg2576/PS1 mice (Sung et al., 2004, Kotilinek et al., 2008). More recent research explored dexibuprofen (DXI) in female APPswe/PS1dE9 mice, showing enhanced non-amyloidogenic processing, reduced inflammation, and improved synaptic plasticity **(**Ettcheto et al., 2021; Guo and Yang, 2024). The mechanism of NSAID action in AD remains unclear, but potential pathways include COX inhibition, PPAR-γ activation, and downregulation of BACE1 transcription (de Oliveira et al., 2021).

#### 5.1.5 Diminutive molecules targeting proinflammatory cytokines

Elevated pro-inflammatory cytokines contribute directly to the progression of AD pathology (Sandhu and Kulka, 2021). MW01–2–151SRM [2-(4-(4-methyl-6-phenylpyridazin-3-yl) piperazin-1-yl) pyrimidine] (which is additionally known as MW-151), a small, aqueously soluble molecule, showed remarkable efficacy in a mouse model of human Aβ-induced damage related to AD. Administration of MW-151 to animals in the initial phase of AD resulted in a reduction of inflammation-promoting cytokine production, neuroglia, and astrocyte triggering in the cerebellum while simultaneously enhancing synaptic plasticity (Bachstetter et al., 2012).

## 6 Preclinical evaluation of anti-neuroinflammatory therapeutics in AD

Various preclinical studies have explored combination therapies targeting different pathological mechanisms in Alzheimer's disease (AD). TREM2 activators, such as the monoclonal antibody Ab18, enhanced microglial phagocytosis of oligomeric amyloid-β (oAβ) and amyloid plaques in 5XFAD mice, reducing Aβ load and improving synaptic and neuronal marker intensity, suggesting enhanced cognitive function (Zhao et al., 2022). These findings, along with others, are summarized in Table 2, which illustrates various preclinical studies involving therapeutic agents used in AD.

**Table 2:** Illustrates the various preclinical studies of the therapeutic agent used in Alzheimer's disease.

**Treatment Type**	**Target**	**Species**	**Mechanism of Action**	**Outcome**	**References**
**TREM2 Activators**	TREM2 receptor	5XFAD mice (Alzheimer’s disease model)	Enhances TREM2 activation, promoting microglial phagocytosis of oAβ and Aβ plaques	The study showed that by improving microglial phagocytosis, the TREM2 agonist monoclonal antibody (Ab18) dramatically decreased the Aβ plaque load. Increased synaptic and neuronal marker intensity also suggested that it enhanced cognitive processes. Additionally, the therapy decreased levels of phosphorylated neurofilament H and tau hyperphosphorylation. Additionally, improved microglial migration and survival toward Aβ plaques were noted, underscoring the potential therapeutic use of modified TREM2-targeting antibodies in Alzheimer's disease.	(Zhao et al., 2022).
**TNF Inhibitors**	TNF-α	Rat	The TREM2 R47H variant impairs microglial Aβ clearance and disrupts the excitatory/inhibitory balance in neurons by enhancing **glutamatergic transmission** and reducing **GABAergic transmission** through elevated **TNF-α** activity. This imbalance increases excitotoxicity, leading to neuronal dysfunction and neurodegeneration.	The TREM2 R47H-induced excitatory/inhibitory imbalance, driven by TNF-α, may represent an early, Aβ-independent mechanism contributing to Alzheimer's disease. Targeting TNF-α could restore neuronal balance and offer therapeutic potential for modifying AD progression.	(Ren et al., 2021).
**IL−1β Inhibitors**	IL−1β	Rat	IFNβ1a exerts its anti-inflammatory effects by binding to the interferon receptor (IFNAR) on glial cells, activating the JAK-STAT signaling pathway. This leads to the production of anti-inflammatory cytokines like IL−10 and suppression of pro-inflammatory cytokines such as IL−6 and IL−1β. IFNβ1a also reduces oxidative stress by increasing superoxide dismutase (SOD1) levels and decreasing reactive oxygen species (ROS) and lipid peroxidation, thereby protecting hippocampal neurons from inflammation-induced damage.	IFNβ1a effectively reverses memory impairment and neuroinflammation in Aβ1–42-injected rats by reducing pro-inflammatory cytokines, enhancing anti-inflammatory responses, and mitigating oxidative stress. Its established efficacy in multiple sclerosis suggests that it may serve as a promising therapeutic strategy for combating inflammation and oxidative stress in Alzheimer’s disease.	(Mudò et al., 2019).
**NSAIDs**	Cyclooxygenase (COX)	Rat	Naproxen, a nonsteroidal anti-inflammatory drug (NSAID), exerts its neuroprotective effects by inhibiting cyclooxygenase (COX)−1 and COX−2, reducing the production of pro-inflammatory prostaglandins. This leads to decreased neuroinflammation and oxidative stress, which helps reduce apoptosis and enhance neurogenesis. Rivastigmine, a cholinesterase inhibitor (ChEI), works by inhibiting acetylcholinesterase (AChE), thereby increasing acetylcholine levels in the synapse and improving cognitive function.	The combination of rivastigmine and naproxen improved neurogenesis and reduced apoptosis in AD rats but did not enhance cognitive function beyond rivastigmine alone. This suggests that naproxen’s neuroprotective effects are more pronounced at the cellular level rather than improving cognitive outcomes.	(Abdel-Aal et al., 2022).
**PDE4 Inhibitors**	PDE4 enzyme		PDE4B encodes an enzyme that hydrolyzes cAMP, a key signaling molecule involved in memory and metabolism. Reduced PDE4B activity (via the Pde4bY358C mutation) increases cAMP levels, which enhances synaptic plasticity and neuronal signaling, thereby improving memory and metabolism in the AppNL-G-F AD model.	Hypomorphic PDE4B activity prevents spatial memory and metabolism deficits in AppNL-G-F mice without reducing Aβ plaque burden, highlighting PDE4B inhibition as a potential therapeutic strategy for AD.	(Armstrong et al., 2024).
**Cannabinoid Receptor 2 (CB2) Agonists**	CB2 receptor	Mouse	Activation of CB2 receptors by the agonist inhibits adenylyl cyclase, reducing cAMP levels and downstream PKA activity. This triggers the MAPK/ERK pathway, enhancing microglial function, reducing inflammation, and promoting Aβ clearance. Modulating CB2 receptors thus shifts microglia toward a neuroprotective state, aiding in Aβ metabolism.	CB2 receptors are expressed in dystrophic neurite-associated microglia, and their modulation influences microglial activity and Aβ metabolism, highlighting CB2 receptors as potential therapeutic targets for Alzheimer’s disease.	(Ruiz de Martín Esteban et al., 2022b).
NSAID	COX−2 inhibitor	Rat	Celecoxib, a selective COX−2 inhibitor, reduces neuroinflammation by inhibiting the overexpression of COX−2, thereby decreasing apoptosis and promoting neurogenesis. Rivastigmine, a cholinesterase inhibitor, enhances cognitive function by increasing acetylcholine levels in the brain. Their combined use targets both inflammation and cognitive decline, improving cellular protection through enhanced neurogenesis and reduced apoptosis.	Although the celecoxib and rivastigmine combination enhances neurogenesis and reduces apoptosis, it does not provide additional cognitive benefits over rivastigmine alone in the aluminum chloride-induced Alzheimer's rat model.	(Abdel-Aal et al., 2021).

## 7 Clinical evaluation of anti-neuroinflammatory therapeutics in AD

Neuroinflammation is a central pathological feature in Alzheimer’s disease (AD), contributing to neuronal damage and cognitive decline. Recent clinical efforts have focused on evaluating therapeutics that modulate inflammatory pathways, particularly targeting microglia, cytokines, and intracellular signaling cascades. Genetic studies have also identified immune-related genes (e.g., TREM2, CD33) associated with increased AD risk, further supporting the role of neuroinflammation in disease development. Fluid biomarkers such as cerebrospinal fluid (CSF) YKL-40, soluble TREM2 (TREM2), and glial fibrillary acidic protein (GFAP) have been linked to different clinical stages of AD, offering potential tools for early diagnosis, disease monitoring, and prognosis (Kiraly et al., 2023b). Table 3 illustrates the various clinical studies of the therapeutic agent used in Alzheimer's disease.

**Table 3:** Various clinical studies of the therapeutic agents used in Alzheimer's disease.

**Compound / Study**	**Treatment Type**	**Key Findings**	**Population**	**Conclusion**	**References**
VG−3927 (Vigil Neuroscience)	Small-molecule TREM2 agonist	Initiated Phase 1 (2023); activates TREM2; brain-penetrant; safety under evaluation	Healthy volunteers	An ongoing clinical trial is to further examine the possibility of VG−3927 as an AD-modifying medication with transformational potential as an oral bioavailable treatment. Initial PKPD investigations have revealed brain penetrance and pharmacological activity in the central nervous system.	(Mirescu et al., 2024).
AL002 (Alector)	Monoclonal antibody TREM2 agonist	Phase 1 showed target engagement and anti-inflammatory response; well-tolerated	64 healthy individuals, 5 early AD patients	These results support AL002's ongoing clinical research for AD and other neurodegenerative conditions where TREM2 activation may be helpful. A phase 2 randomised, double-blind, placebo-controlled study is presently testing AL002 in early AD.	(Long et al., 2024).
RESIST Study (TNFi vs. csDMARDs)	TNF α Inhibitor	No significant difference in cognitive outcomes (FCSRT, Moca) at 6 and 12 months	130 Observational cohort study carried patients with MCI	No cognitive benefit observed; longer follow-up needed	(Marr et al., 2023)
NSAIDs	Aspirin, Ibuprofen	A significant association was observed between NSAID use and improved cognitive status.	cross-sectional and longitudinal studies.	NSAID usage, especially ibuprofen, may offer neuroprotective benefits through anti-inflammatory pathways and could reduce Alzheimer's disease risk when administered early.	(Morris et al., 2020).
Rofumilast	PDE4	Multiple cognitive domains, quality of life, well-being, and daily functioning (assessed via participant and caregiver interviews)	Double-blind, randomised, placebo-controlled, between-subjects clinical trial. Three arms: Placebo, 50 μg roflumilast, and 100 μg roflumilast. Treatment duration: 24 weeks.	The study aims to establish proof-of-concept that PDE4 inhibition via roflumilast can improve cognitive function and daily life in early-stage AD patients, supporting its further development as a therapeutic agent for neurodegenerative disorders.	(Possemis et al., 2024).
Cannabidiol	Cannabinoid Receptor 2 (CB2) Agonists	CBD at 600 mg/day was well-tolerated with no serious adverse events or withdrawals among Alzheimer’s patients with BPSD.	Randomised into two groups: CBD group: 600 mg daily for 6 weeks Placebo group: daily for 6 Weeks	CBD shows promise as a safe and acceptable treatment for BPSD in AD, warranting further investigation into its clinical efficacy.	(Vuic et al., 2022).
Etanercept (25–50 mg) (Perispinal Administration)	TNF-α inhibitor	Open-label 6-month study showed significant cognitive improvements (MMSE ↑2.13, ADAS-Cog ↓5.48, SIB ↑16.6	15 patients with moderate AD	Suggests potential cognitive benefits; larger RCTS needed to confirm efficacy	(Edward Tobinick, 2006).

## 8 Future prospects of targeting inflammation in AD

Current findings highlight the multifaceted nature of neuroinflammation in Alzheimer’s disease, with multiple signaling cascades including cytokine release and endocannabinoid modulation contributing to pathogenesis. While individual targeting of pathways such as IL-1β or CB2 has shown promise in preclinical models, the complexity of neuroimmune signaling suggests that monotherapy may be insufficient for sustained disease modification. Future trials should explore combination immunotherapies targeting both IL-1β and CB2 pathways in genetically stratified AD models, particularly those carrying risk alleles such as *APOE4* or *TREM2*. This dual-target strategy may offer synergistic benefits by simultaneously modulating pro-inflammatory cytokines and enhancing microglial phagocytic function.

## 9 Conclusion

The intricate interplay between neuroinflammation and Alzheimer's disease pathogenesis represents a paradigm shift in our understanding of neurodegeneration. Beyond the traditional focus on Aβ plaques and tau tangles, this review highlights how dysregulated immune responses mediated by microglia, astrocytes, cytokines, and inflammasomes act as critical amplifiers of neuronal damage. Targeting these immunological pathways provides a promising therapeutic avenue, particularly in light of the limited success of conventional anti-Aβ and anti-tau interventions. By synthesizing current mechanistic insights with emerging preclinical and clinical data on anti-inflammatory strategies, this review underscores the need for an integrated, systems-level approach to AD therapy. Future success will likely depend on the development of precision-based treatments that account for individual immune signatures and the temporal dynamics of neuroinflammatory processes. This analytical perspective contributes to a more nuanced understanding of AD and opens the door for innovative therapeutic paradigms grounded in immunomodulation.

## CRediT authorship contribution statement

**Pritam Kamila:** Writing – original draft, Methodology, Data curation, Conceptualization. **Bhupendra Gopalbhai Prajapati:** Writing – review & editing, Validation, Supervision, Resources, Project administration, Formal analysis. **Singh S. Ankul:** Writing – original draft, Methodology, Data curation. **S Sowmiya:** Writing – original draft, Data curation. **Ria Dutta:** Writing – original draft, Visualization, Validation, Supervision, Resources. **Priyanka Chakraborty:** Writing – original draft, Resources, Formal analysis, Data curation. **Sailee Chowdhury:** Writing – review & editing, Writing – original draft, Supervision, Project administration, Data curation, Conceptualization. **Koyel Kar:** Writing – original draft, Visualization, Resources, Methodology.

## Funding

None.

## Conflicts of Interest

The authors declare that they have no known competing financial interests or personal relationships that could have appeared to influence the work reported in this paper.

## References

[bib1] Abdel-Aal R.A., Hussein O.A., Elsaady R.G., Abdelzaher L.A. (2022). Naproxen as a potential candidate for promoting rivastigmine anti-Alzheimer activity against aluminum chloride-prompted Alzheimer’s-like disease in rats; neurogenesis and apoptosis modulation as a possible underlying mechanism. Eur. J. Pharmacol..

[bib2] Abdel-Aal R., Hussein O., Elsaady R., Abdelzaher L. (2021). Celecoxib effect on rivastigmine anti-Alzheimer activity against aluminum chloride-induced neurobehavioral deficits as a rat model of Alzheimer’s disease; novel perspectives for an old drug. J. Med. Life Sci..

[bib3] Adamu A., Li S., Gao F., Xue G. (2024). The role of neuroinflammation in neurodegenerative diseases: current understanding and future therapeutic targets. Front. Aging Neurosci..

[bib4] Armstrong P., Güngör H., Anongjanya P., Tweedy C., Parkin E., Johnston J. (2024). Protective effect of PDE4B subtype-specific inhibition in an App knock-in mouse model for Alzheimer’s disease. Neuropsychopharmacology.

[bib5] Azargoonjahromi A. (2023). Dual role of nitric oxide in Alzheimer’s disease. Nitric Oxide.

[bib6] Bachstetter A.D., Norris C.M., Sompol P., Wilcock D.M., Goulding D., Neltner J.H. (2012). Early stage drug treatment that normalizes proinflammatory cytokine production attenuates synaptic dysfunction in a mouse model that exhibits age-dependent progression of alzheimer’s disease-related pathology. J. Neurosci..

[bib7] Balez R., Ooi L. (2016). Getting to no alzheimer’s disease: neuroprotection versus neurotoxicity mediated by nitric oxide. Oxid. Med. Cell. Longev..

[bib8] Bejanin A., Schonhaut D.R., La Joie R., Kramer J.H., Baker S.L., Sosa N. (2017). Tau pathology and neurodegeneration contribute to cognitive impairment in Alzheimer’s disease. Brain.

[bib9] Bekris L.M., Khrestian M., Dyne E., Shao Y., Pillai J.A., Rao S.M. (2018). Soluble TREM2 and biomarkers of central and peripheral inflammation in neurodegenerative disease. J. Neuroimmunol..

[bib10] Belenguer P., Duarte J.M.N., Schuck P.F., Ferreira G.C. (2019). Mitochondria and the Brain: bioenergetics and beyond. Neurotox. Res..

[bib11] Benito C., Tolón R.M., Pazos M.R., Núñez E., Castillo A.I., Romero J. (2008). Cannabinoid CB 2 receptors in human brain inflammation. Br. J. Pharmacol..

[bib12] Bie B., Wu J., Foss J.F., Naguib M. (2018). An overview of the cannabinoid type 2 receptor system and its therapeutic potential. Curr. Opin. Anaesthesiol..

[bib13] Brand A.L., Lawler P.E., Bollinger J.G., Li Y., Schindler S.E., Li M. (2022). The performance of plasma amyloid beta measurements in identifying amyloid plaques in Alzheimer’s disease: a literature review. Alzheimers Res. Ther..

[bib14] Chakrabarty P., Li A., Ceballos-Diaz C., Eddy J.A., Funk C.C., Moore B. (2015). IL-10 Alters Immunoproteostasis in APP Mice, increasing plaque burden and worsening cognitive behavior. Neuron..

[bib15] Chen Y., Yu Y. (2023). Tau and neuroinflammation in Alzheimer’s disease: interplay mechanisms and clinical translation. J. Neuroinflamm..

[bib16] Chen H., Zeng Y., Wang D., Li Y., Xing J., Zeng Y. (2024). Neuroinflammation of microglial regulation in alzheimer’s disease: therapeutic approaches. Molecules.

[bib17] Chowdhury S., Kar K., Mazumder R. (2024). Exploration of different strategies of nanoencapsulation of bioactive compounds and their ensuing approaches. Future J. Pharm. Sci..

[bib18] Colonna M., Butovsky O. (2017). Microglia function in the central nervous system during health and neurodegeneration. Annu. Rev. Immunol..

[bib19] Cummings J., Lee G., Zhong K., Fonseca J., Taghva K. (2021). Alzheimer's disease drug development pipeline: 2021. Alzheimer Dement. Transl. Res. Clin. Interv..

[bib20] Czirr E., Wyss-Coray T. (2012). The immunology of neurodegeneration. J. Clin. Investig..

[bib21] Dani M., Wood M., Mizoguchi R., Fan Z., Walker Z., Morgan R. (2018). Microglial activation correlates in vivo with both tau and amyloid in Alzheimer’s disease. Brain.

[bib22] Decourt B., Lahiri D.K., Sabbagh M.N. (2017). Targeting Tumor Necrosis Factor Alpha for Alzheimer’s disease. Curr. Alzheimer's Res..

[bib23] Detrait E.R., Danis B., Lamberty Y., Foerch P. (2014). Peripheral administration of an anti-TNF-α receptor fusion protein counteracts the amyloid induced elevation of hippocampal TNF-α levels and memory deficits in mice. Neurochem. Int..

[bib24] DeTure M.A., Dickson D.W. (2019). The neuropathological diagnosis of Alzheimer’s disease. Mol. Neurodegener..

[bib25] Dionisio-Santos D.A., Olschowka J.A., O’Banion M.K. (2019). Exploiting microglial and peripheral immune cell crosstalk to treat Alzheimer’s disease. J. Neuroinflamm..

[bib26] Domingues C., da Cruz e Silva O.A.B., Henriques A.G. (2017). Impact of cytokines and chemokines on Alzheimer’s disease neuropathological hallmarks. Curr. Alzheimer's Res..

[bib27] Dong Y., Fischer R., Naudé P.J.W., Maier O., Nyakas C., Duffey M. (2016). Essential protective role of tumor necrosis factor receptor 2 in neurodegeneration. Proc. Natl. Acad. Sci..

[bib28] Dubois B., Hampel H., Feldman H.H., Scheltens P., Aisen P., Andrieu S. (2016). Preclinical Alzheimer’s disease: definition, natural history, and diagnostic criteria. Alzheimer’s Dement..

[bib29] Edward Tobinick (2006). 1 HG 2, AW 3, HC 4. TNF-alpha modulation for treatment of Alzheimer’s disease: a 6-month pilot study. MedGenMed.

[bib30] Esposito G., Iuvone T., Savani C., Scuderi C., De Filippis D., Papa M. (2007). Opposing control of cannabinoid receptor stimulation on amyloid-β-Induced reactive gliosis: in vitro and in vivo evidence. J. Pharmacol. Exp. Ther..

[bib31] Ettcheto M., Sánchez-Lopez E., Cano A., Carrasco M., Herrera K., Manzine P.R. (2021). Dexibuprofen ameliorates peripheral and central risk factors associated with Alzheimer’s disease in metabolically stressed APPswe/PS1dE9 mice. Cell Biosci..

[bib32] Fisher A. (2012). Cholinergic modulation of amyloid precursor protein processing with emphasis on M1 muscarinic receptor: perspectives and challenges in treatment of Alzheimer’s disease. J. Neurochem..

[bib33] Forlenza O.V., Diniz B.S., Talib L.L., Mendonça V.A., Ojopi E.B., Gattaz W.F. (2009). Increased Serum IL-1β Level in Alzheimer’s Disease and Mild Cognitive Impairment. Dement. Geriatr. Cogn. Disord..

[bib34] Galimberti D., Fenoglio C., Scarpini E. (2008). Inflammation in neurodegenerative disorders: friend or foe?. Curr. Aging Sci..

[bib35] Ghosh M., Xu Y., Pearse D.D. (2016). Cyclic AMP is a key regulator of M1 to M2a phenotypic conversion of microglia in the presence of Th2 cytokines. J. Neuroinflamm..

[bib36] Graybeal J.J., Bozzelli P.L., Graybeal L.L., Groeber C.M., McKnight P.E., Cox D.N. (2014). Human apoe ε4 alters circadian rhythm activity, il-1β, and gfap in crnd8 mice. J. Alzheimer’s Dis..

[bib37] Guerreiro R., Wojtas A., Bras J., Carrasquillo M., Rogaeva E., Majounie E. (2013). TREM2 Variants in Alzheimer’s Disease. N. Engl. J. Med..

[bib38] Guo S., Wang H., Yin Y. (2022). Microglia polarization From M1 to M2 in Neurodegenerative Diseases. Front. Aging Neurosci..

[bib39] Guo S., Yang J. (2024). Bayesian genome-wide TWAS with reference transcriptomic data of brain and blood tissues identified 141 risk genes for Alzheimer’s disease dementia. Alzheimer's Res. Ther..

[bib40] Hanisch U.K., Kettenmann H. (2007). Microglia: active sensor and versatile effector cells in the normal and pathologic brain. Nat. Neurosci..

[bib41] Hardy J., Selkoe D.J. (2002). The amyloid hypothesis of Alzheimer’s disease: progress and problems on the road to therapeutics. Science.

[bib42] Hickman S.E., Allison E.K., El Khoury J. (2008). Microglial dysfunction and defective β-amyloid clearance pathways in aging Alzheimer’s disease mice. J. Neurosci..

[bib43] Hickman S.E., Kingery N.D., Ohsumi T.K., Borowsky M.L., Wang L. chong, Means T.K. (2013). The microglial sensome revealed by direct RNA sequencing. Nat. Neurosci..

[bib44] Ito M., Komai K., Mise-Omata S., Iizuka-Koga M., Noguchi Y., Kondo T. (2019). Brain regulatory T cells suppress astrogliosis and potentiate neurological recovery. Nature.

[bib45] Jantzen P.T., Connor K.E., DiCarlo G., Wenk G.L., Wallace J.L., Rojiani A.M. (2002). Microglial activation and β-amyloid deposit reduction caused by a nitric oxide-releasing nonsteroidal anti-inflammatory drug in amyloid precursor protein plus presenilin-1 transgenic mice. J. Neurosci..

[bib46] Ji K., Akgul G., Wollmuth L.P., Tsirka S.E., Dunaevsky A. (2013).

[bib47] Kang S., Gim J., Lee J., Gunasekaran T.I., Choi K.Y., Lee J.J. (2021). Potential novel genes for late-onset Alzheimer’s disease in east-asian descent identified by apoe-stratified genome-wide association study. J. Alzheimer’s Dis..

[bib48] Karlawish J., Jack C.R., Rocca W.A., Snyder H.M., Carrillo M.C. (2017). Alzheimer’s disease: the next frontier special report 2017. Alzheimer’s Dement..

[bib49] Kepp K.P., Robakis N.K., Høilund-Carlsen P.F., Sensi S.L., Vissel B. (2023). The amyloid cascade hypothesis: an updated critical review. Brain.

[bib50] Keren-Shaul H., Spinrad A., Weiner A., Matcovitch-Natan O., Dvir-Szternfeld R., Ulland T.K. (2017). A unique microglia type associated with restricting development of Alzheimer’s disease. Cell.

[bib51] Khatri N., Man H.Y. (2013). Synaptic activity and bioenergy homeostasis: implications in brain trauma and neurodegenerative diseases. Front. Neurol..

[bib52] Kim T.S., Lim H.K., Lee J.Y., Kim D.J., Park S., Lee C. (2008). Changes in the levels of plasma soluble fractalkine in patients with mild cognitive impairment and Alzheimer’s disease. Neurosci. Lett..

[bib53] Kiraly M., Foss J.F., Giordano T. (2023). Neuroinflammation, Its role in Alzheimer’s disease and therapeutic strategies. J. Prev. Alzheimer's Dis..

[bib54] Kiraly M., Foss J.F., Giordano T. (2023). Neuroinflammation, Its role in Alzheimer’s disease and therapeutic strategies. J. Prev. Alzheimer's Dis..

[bib55] Kopke E., Tung Y.C., Shaikh S., Alejandra del C., Alonso K.I. (1993). Grundke-Iqba and I. microtubule-associated Protein Tau. J. Biol. Chem..

[bib56] Kotilinek L.A., Westerman M.A., Wang Q., Panizzon K., Lim G.P., Simonyi A. (2008). Cyclooxygenase-2 inhibition improves amyloid-β-mediated suppression of memory and synaptic plasticity. Brain.

[bib57] Krasemann S., Madore C., Cialic R., Baufeld C., Calcagno N., El Fatimy R. (2017). The TREM2-APOE pathway drives the transcriptional phenotype of dysfunctional microglia in neurodegenerative diseases. Immunity.

[bib58] Kreisl W.C., Lyoo C.H., McGwier M., Snow J., Jenko K.J., Kimura N. (2013). In vivo radioligand binding to translocator protein correlates with severity of Alzheimer’s disease. Brain.

[bib59] Lannfelt L., Relkin N.R., Siemers E.R. (2014). Amyloid-ß-directed immunotherapy for Alzheimer’s disease. J. Intern. Med..

[bib60] Lécuyer M.A., Kebir H., Prat A. (2016). Glial influences on BBB functions and molecular players in immune cell trafficking. Biochim. Biophys. Acta Mol. Basis Dis..

[bib61] Lee C.Y.D., Daggett A., Gu X., Jiang L.L., Langfelder P., Li X. (2018). Elevated TREM2 gene dosage reprograms microglia responsivity and Ameliorates pathological phenotypes in alzheimer’s disease models. Neuron.

[bib62] Leng F., Edison P. (2021). Neuroinflammation and microglial activation in Alzheimer disease: where do we go from here?. Nat. Rev. Neurol..

[bib63] Leng F., Edison P. (2021). Neuroinflammation and microglial activation in Alzheimer disease: where do we go from here?. Nat. Rev. Neurol..

[bib64] Leng F., Edison P. (2021). Neuroinflammation and microglial activation in Alzheimer disease: where do we go from here?. Nat. Rev. Neurol..

[bib65] Liddelow S.A., Guttenplan K.A., Clarke L.E., Bennett F.C., Bohlen C.J., Schirmer L. (2017). Neurotoxic reactive astrocytes are induced by activated microglia. Nature.

[bib66] Liddelow S.A., Guttenplan K.A., Clarke L.E., Bennett F.C., Bohlen C.J., Schirmer L. (2017). Neurotoxic reactive astrocytes are induced by activated microglia. Nature.

[bib67] Lim G.P., Yang F., Chu T., Chen P., Beech W., Teter B. (2000). Ibuprofen suppresses plaque pathology and inflammation in a mouse model for alzheimer’s disease. J. Neurosci..

[bib68] Lista S., Imbimbo B.P., Grasso M., Fidilio A., Emanuele E., Minoretti P. (2024). Tracking neuroinflammatory biomarkers in Alzheimer’s disease: a strategy for individualized therapeutic approaches?. J. Neuroinflamm..

[bib69] Li R., Wang X., He P. (2021). The most prevalent rare coding variants of TREM2 conferring risk of Alzheimer’s disease: a systematic review and meta-analysis. Exp. Ther. Med..

[bib70] Li Y., Xu H., Wang H., Yang K., Luan J., Wang S. (2023). TREM2: potential therapeutic targeting of microglia for Alzheimer’s disease. Biomed. Pharmacother..

[bib71] Li H., Yu W., Zheng X., Zhu Z. (2025). TREM1—Microglia crosstalk: neurocognitive disorders. Brain Res. Bull..

[bib72] Long H., Simmons A., Mayorga A., Burgess B., Nguyen T., Budda B. (2024). Preclinical and first-in-human evaluation of AL002, a novel TREM2 agonistic antibody for Alzheimer’s disease. Alzheimer's Res. Ther..

[bib73] Luca A., Calandra C., Luca M. (2018). Molecular bases of Alzheimer’s disease and neurodegeneration: the role of neuroglia. Aging Dis..

[bib74] Lyman M., Lloyd D.G., Ji X., Vizcaychipi M.P., Ma D. (2014). Neuroinflammation: The role and consequences. Neurosci. Res..

[bib75] MacEwan D.J. (2002). TNF ligands and receptors a matter of life and death. Br. J. Pharmacol..

[bib76] Magham S.V., Thaggikuppe krishnamurthy P., Shaji N., Mani L., Balasubramanian S. (2021). Cannabinoid receptor 2 selective agonists and Alzheimer’s disease: an insight into the therapeutic potentials. J. Neurosci. Res..

[bib77] Malko P., Syed Mortadza S.A., McWilliam J., Jiang L.H. (2019). TRPM2 channel in microglia as a new player in neuroinflammation associated with a spectrum of central nervous system pathologies. Front. Pharmacol..

[bib78] Marr C, McDowell B, Holmes C, Edwards CJ, Cardwell C, McHenry M (2023). The RESIST study: do TNF inhibitors protect against cognitive decline in rheumatoid arthritis patients with mild cognitive impairment?. Alzheimer’s Dement..

[bib79] Mathys H., Adaikkan C., Gao F., Young J.Z., Manet E., Hemberg M. (2017). Temporal tracking of microglia activation in neurodegeneration at single-cell resolution. Cell Rep..

[bib80] Mirescu C., Dejanovic B., Larson K.C., Kiragasi B., Gergits F.W., Figley M.D. (2024). Pharmacological and functional characterization of the first small molecule TREM2 agonist, VG-3927, for the treatment of Alzheimer’s disease. Alzheimer’s Dement..

[bib81] Mishra A., Kim H.J., Shin A.H., Thayer S.A. (2012). Synapse loss induced by interleukin-1β requires pre and post-synaptic mechanisms. J. Neuroimmune Pharmacol..

[bib82] Mohandas E., Rajmohan V., Raghunath B. (2009). Neurobiology of Alzheimer′s disease. Indian J. Psychiatry..

[bib83] Monteiro A.R., Barbosa D.J., Remião F., Silva R. (2023). Alzheimer’s disease: Insights and new prospects in disease pathophysiology, biomarkers and disease-modifying drugs. Biochem. Pharmacol..

[bib84] Morris R., Armbruster K., Silva J., Widell D.J., Cheng F. (2020). The association between the usage of non-steroidal anti-inflammatory drugs and cognitive status: analysis of longitudinal and cross-sectional studies from the global Alzheimer’s association interactive network and transcriptomic data. Brain Sci..

[bib85] Mudò G., Frinchi M., Nuzzo D., Scaduto P., Plescia F., Massenti M.F. (2019). Anti-inflammatory and cognitive effects of interferon-β1a (IFNβ1a) in a rat model of Alzheimer’s disease. J. Neuroinflamm..

[bib86] Novoa C., Salazar P., Cisternas P., Gherardelli C., Vera-Salazar R., Zolezzi J.M. (2022). Inflammation context in Alzheimer’s disease, a relationship intricate to define. Biol. Res..

[bib87] Núñez E., Benito C., Pazos M.R., Barbachano A., Fajardo O., González S. (2004). Cannabinoid CB 2 receptors are expressed by perivascular microglial cells in the human brain: An immunohistochemical study. Synapse.

[bib88] Ogunmokun G., Dewanjee S., Chakraborty P., Valupadas C., Chaudhary A., Kolli V. (2021). The potential role of cytokines and growth factors in the pathogenesis of Alzheimer’s disease. Cells.

[bib89] Ogunmokun G., Dewanjee S., Chakraborty P., Valupadas C., Chaudhary A., Kolli V. (2021). The potential role of cytokines and growth factors in the pathogenesis of Alzheimer’s disease. Cells.

[bib90] de Oliveira J., Kucharska E., Garcez M.L., Rodrigues M.S., Quevedo J., Moreno-Gonzalez I. (2021). Inflammatory cascade in Alzheimer’s disease pathogenesis: a review of experimental findings. Cells.

[bib91] Onyango I.G., Dennis J., Khan S.M. (2016). Mitochondrial dysfunction in Alzheimer’s disease and the rationale for bioenergetics based therapies. Aging Dis..

[bib92] Onyango I.G., Jauregui G.V., Čarná M., Bennett J.P., Stokin G.B. (2021). Neuroinflammation in Alzheimer’s disease. Biomedicines..

[bib93] Pérez M.J., Jara C., Quintanilla R.A. (2018). Contribution of tau pathology to mitochondrial impairment in neurodegeneration. Front. Neurosci..

[bib94] Picón-Pagès P., Garcia-Buendia J., Muñoz F.J. (2019). Functions and dysfunctions of nitric oxide in brain. Biochim. Biophys. Acta Mol. Basis Dis..

[bib95] Possemis N., Verhey F., Prickaerts J., Blokland A., Ramakers I. (2024). A proof of concept phase II study with the PDE-4 inhibitor roflumilast in patients with mild cognitive impairment or mild Alzheimer’s disease dementia (ROMEMA): study protocol of a double-blind, randomized, placebo-controlled, between-subjects trial. Trials.

[bib96] Price B.R., Sudduth T.L., Weekman E.M., Johnson S., Hawthorne D., Woolums A. (2020). Therapeutic Trem2 activation ameliorates amyloid-beta deposition and improves cognition in the 5XFAD model of amyloid deposition. J. Neuroinflamm..

[bib97] Prince M., Ali G.C., Guerchet M., Prina A.M., Albanese E., Wu Y.T. (2016). Recent global trends in the prevalence and incidence of dementia, and survival with dementia. Alzheimer's Res. Ther..

[bib98] Reardon S. (2023). Alzheimer’s drug donanemab helps most when taken at earliest disease stage, study finds. Nature.

[bib99] Reed T., Perluigi M., Sultana R., Pierce W.M., Klein J.B., Turner D.M. (2008). Redox proteomic identification of 4-Hydroxy-2-nonenal-modified brain proteins in amnestic mild cognitive impairment: Insight into the role of lipid peroxidation in the progression and pathogenesis of Alzheimer’s disease. Neurobiol. Dis..

[bib100] Ren S., Breuillaud L., Yao W., Yin T., Norris K.A., Zehntner S.P. (2021). TNF-α–mediated reduction in inhibitory neurotransmission precedes sporadic Alzheimer’s disease pathology in young Trem2 rats. J. Biol. Chem..

[bib101] Ruiz de Martín Esteban S., Benito-Cuesta I., Terradillos I., Martínez-Relimpio A.M., Arnanz M.A., Ruiz-Pérez G. (2022). Cannabinoid CB2 receptors modulate microglia function and amyloid dynamics in a mouse model of Alzheimer’s disease. Front. Pharmacol..

[bib102] Ruiz de Martín Esteban S., Benito-Cuesta I., Terradillos I., Martínez-Relimpio A.M., Arnanz M.A., Ruiz-Pérez G. (2022). Cannabinoid CB2 receptors modulate microglia function and amyloid dynamics in a mouse model of Alzheimer’s Disease. Front. Pharmacol..

[bib103] Sanchez-Mejias E., Navarro V., Jimenez S., Sanchez-Mico M., Sanchez-Varo R., Nuñez-Diaz C. (2016). Soluble phospho-tau from Alzheimer’s disease hippocampus drives microglial degeneration. Acta Neuropathol..

[bib104] Sandhu J.K., Kulka M. (2021). Decoding mast cell-microglia communication in neurodegenerative diseases. Int. J. Mol. Sci..

[bib105] Selkoe D.J. (2008). Soluble oligomers of the amyloid β-protein impair synaptic plasticity and behavior. Behav. Brain Res..

[bib106] Shi Q., Chowdhury S., Ma R., Le K.X., Hong S., Caldarone B.J. (2017). Complement C3 deficiency protects against neurodegeneration in aged plaque-rich APP/PS1 mice. Sci. Transl. Med..

[bib107] Shue F., White L.J., Hendrix R., Ulrich J., Henson R.L., Knight W. (2024). CSF biomarkers of immune activation and Alzheimer’s disease for predicting cognitive impairment risk in the elderly. Sci. Adv..

[bib108] Song W.M., Joshita S., Zhou Y., Ulland T.K., Gilfillan S., Colonna M. (2018). Humanized TREM2 mice reveal microglia-intrinsic and -extrinsic effects of R47H polymorphism. J. Exp. Med..

[bib109] Spiers J.G., Chen H.J.C., Bourgognon J.M., Steinert J.R. (2019). Dysregulation of stress systems and nitric oxide signaling underlies neuronal dysfunction in Alzheimer’s disease. Free Radic. Biol. Med..

[bib110] Stebbing M.J., Cottee J.M., Rana I. (2015). The role of ion channels in microglial activation and proliferation a complex interplay between ligand-gated ion channels, K+ channels, and intracellular Ca2+. Front Immunol..

[bib111] Sung S., Yang H., Uryu K., Lee E.B., Zhao L., Shineman D. (2004). Modulation of Nuclear Factor-κb Activity by Indomethacin Influences Aβ Levels But Not Aβ precursor protein metabolism in a model of Alzheimer’s disease. Am. J. Pathol..

[bib112] Sun Z., Zhang X., So K.F., Jiang W., Chiu K. (2024). Targeting Microglia in Alzheimer’s disease: pathogenesis and potential therapeutic strategies. Biomolecules.

[bib113] Szmydynger-Chodobska J., Strazielle N., Zink B.J., Ghersi-Egea J.F., Chodobski A. (2009). The role of the choroid plexus in neutrophil invasion after traumatic brain injury. J. Cereb. Blood Flow Metab..

[bib114] Toader C., Tataru C.P., Munteanu O., Serban M., Covache-Busuioc R.A., Ciurea A.V. (2024). Decoding neurodegeneration: a review of molecular mechanisms and therapeutic advances in Alzheimer’s, parkinson’s, and ALS. Int. J. Mol. Sci..

[bib115] Tracy T.E., Madero-Pérez J., Swaney D.L., Chang T.S., Moritz M., Konrad C. (2022). Tau interactome maps synaptic and mitochondrial processes associated with neurodegeneration. Cell.

[bib116] Venegas C., Kumar S., Franklin B.S., Dierkes T., Brinkschulte R., Tejera D. (2017). Microglia-derived ASC specks cross-seed amyloid-β in Alzheimer’s disease. Nature.

[bib117] Venturelli M., Pedrinolla A., Boscolo Galazzo I., Fonte C., Smania N., Tamburin S. (2018). Impact of nitric oxide bioavailability on the progressive cerebral and peripheral circulatory impairments during aging and Alzheimer’s disease. Front. Physiol..

[bib118] vom Berg J., Prokop S., Miller K.R., Obst J., Kälin R.E., Lopategui-Cabezas I. (2012). Inhibition of IL-12/IL-23 signaling reduces Alzheimer’s disease–like pathology and cognitive decline. Nat. Med..

[bib119] vom Berg J., Prokop S., Miller K.R., Obst J., Kälin R.E., Lopategui-Cabezas I. (2012). Inhibition of IL-12/IL-23 signaling reduces Alzheimer’s disease like pathology and cognitive decline. Nat. Med..

[bib120] Vuic B., Milos T., Tudor L., Konjevod M., Nikolac Perkovic M., Jazvinscak Jembrek M. (2022). Cannabinoid CB2 receptors in neurodegenerative proteinopathies: new insights and therapeutic potential. Biomedicines.

[bib121] Wang S., Colonna M. (2023). The microglial immunoreceptor tyrosine-based motif-Syk signaling pathway is a promising target of immunotherapy for Alzheimer’s disease. Clin. Transl. Med..

[bib122] Wang Y., Mandelkow E. (2016). Tau in physiology and pathology. Nat. Rev. Neurosci..

[bib123] Wang S., Mustafa M., Yuede C.M., Salazar S.V., Kong P., Long H. (2020). Anti-human TREM2 induces microglia proliferation and reduces pathology in an Alzheimer’s disease model. J. Exp. Med..

[bib124] Wang S., Mustafa M., Yuede C.M., Salazar S.V., Kong P., Long H. (2020). Anti-human TREM2 induces microglia proliferation and reduces pathology in an Alzheimer’s disease model. J. Exp. Med..

[bib125] Wang H., Zong Y., Zhu L., Wang W., Han Y. (2023). Chemokines in patients with Alzheimer’s disease: A meta-analysis. Front. Aging Neurosci..

[bib126] Westin K., Buchhave P., Nielsen H., Minthon L., Janciauskiene S., Hansson O., Ginsberg S.D. (2012).

[bib127] Wojcieszak J., Kuczyńska K., Zawilska J.B. (2022). Role of chemokines in the development and progression of Alzheimer’s disease. J. Mol. Neurosci..

[bib128] Wu J., Bie B., Yang H., Xu J.J., Brown D.L., Naguib M. (2013). Activation of the CB2 receptor system reverses amyloid-induced memory deficiency. Neurobiol. Aging..

[bib129] Wu J., Hocevar M., Foss J.F., Bie B., Naguib M. (2017). Activation of CB2 receptor system restores cognitive capacity and hippocampal Sox2 expression in a transgenic mouse model of Alzheimer’s disease. Eur. J. Pharmacol..

[bib130] Wu J., Hocevar M., Foss J.F., Bie B., Naguib M. (2017). Activation of CB2 receptor system restores cognitive capacity and hippocampal Sox2 expression in a transgenic mouse model of Alzheimer’s disease. Eur. J. Pharmacol..

[bib131] Xiong L.L., Xue L.L., Du R.L., Niu R.Z., Chen L., Chen J. (2021). Single-cell RNA sequencing reveals B cell–related molecular biomarkers for Alzheimer’s disease. Exp. Mol. Med..

[bib132] Yang X., Wang J., Jia X., Yang Y., Fang Y., Ying X. (2025). Microglial polarization in Alzheimer’s disease: mechanisms, implications, and therapeutic opportunities. J. Alzheimer’s Dis..

[bib133] Yang S.H. (2019). Cellular and molecular mediators of neuroinflammation in Alzheimer disease. Int. Neurourol. J..

[bib134] Zenaro E., Pietronigro E., Bianca V.Della, Piacentino G., Marongiu L., Budui S. (2015). Neutrophils promote Alzheimer’s disease–like pathology and cognitive decline via LFA-1 integrin. Nat. Med..

[bib135] Zhang Y., Fung I.T.H., Sankar P., Chen X., Robison L.S., Ye L. (2020). Depletion of NK cells improves cognitive function in the Alzheimer disease mouse model. J. Immunol..

[bib136] Zhang L., Xiang X., Li Y., Bu G., Chen X.F. (2025). TREM2 and sTREM2 in Alzheimer’s disease: from mechanisms to therapies. Mol. Neurodegener..

[bib137] Zhao P., Xu Y., Jiang L., Fan X., Li L., Li X. (2022). A tetravalent TREM2 agonistic antibody reduced amyloid pathology in a mouse model of Alzheimer’s disease. Sci. Transl. Med..

[bib138] Zhong L., Xu Y., Zhuo R., Wang T., Wang K., Huang R. (2019). Soluble TREM2 ameliorates pathological phenotypes by modulating microglial functions in an Alzheimer’s disease model. Nat. Commun..

[bib139] Zhu Xi-Chen, Tan Lan, Jiang Teng, Tan Meng-Shan, Zhang Wei, Yu Jin-Tai (2014). Association of IL-12A and IL-12B polymorphisms with Alzheimer’s disease susceptibility in a Han Chinese population. J. Neuroimmunol..

[bib140] Zou S., Kumar U. (2018). Cannabinoid Receptors and the endocannabinoid system: signaling and function in the central nervous system. Int. J. Mol. Sci..

